# Regulation of nucleosome positioning by a CHD Type III chromatin remodeler and its relationship to developmental gene expression in *Dictyostelium*

**DOI:** 10.1101/gr.216309.116

**Published:** 2017-04

**Authors:** James L. Platt, Nicholas A. Kent, Alan R. Kimmel, Adrian J. Harwood

**Affiliations:** 1 School of Biosciences, Cardiff University, Cardiff, CF10 3AX, United Kingdom;; 2 Laboratory of Cellular and Developmental Biology, National Institutes of Diabetes and Digestive and Kidney Diseases, National Institutes of Health, Bethesda, Maryland 20892, USA;; 3 Neuroscience and Mental Health Research Institute, Cardiff University, Cardiff, CF24 4HQ, United Kingdom

## Abstract

Nucleosome placement and repositioning can direct transcription of individual genes; however, the precise interactions of these events are complex and largely unresolved at the whole-genome level. The Chromodomain-Helicase-DNA binding (CHD) Type III proteins are a subfamily of SWI2/SNF2 proteins that control nucleosome positioning and are associated with several complex human disorders, including CHARGE syndrome and autism. Type III CHDs are required for multicellular development of animals and *Dictyostelium* but are absent in plants and yeast. These CHDs can mediate nucleosome translocation in vitro, but their in vivo mechanism is unknown. Here, we use genome-wide analysis of nucleosome positioning and transcription profiling to investigate the in vivo relationship between nucleosome positioning and gene expression during development of wild-type (WT) *Dictyostelium* and mutant cells lacking ChdC, a Type III CHD protein ortholog. We demonstrate major nucleosome positional changes associated with developmental gene regulation in WT. Loss of *chdC* caused an increase of intragenic nucleosome spacing and misregulation of gene expression, affecting ∼50% of the genes that are repositioned during WT development. These analyses demonstrate active nucleosome repositioning during *Dictyostelium* multicellular development, establish an in vivo function of CHD Type III chromatin remodeling proteins in this process, and reveal the detailed relationship between nucleosome positioning and gene regulation, as cells transition between developmental states.

Nucleosomes are the basic unit of chromatin structure, with relative nucleosome positioning often influencing higher order organization (Li and Reinberg 2011). Nucleosomes are not stably fixed at DNA locales but may be repositioned in response to intrinsic and extrinsic cues (Ryan and Owen-Hughes 2011). As sequence-specific occupancy by nucleosomes or transcription factors can be mutually exclusive, regulated nucleosome positioning may directly alter transcriptional activity (Struhl and Segal 2013). However, at the global level, the dynamics of nucleosome positioning and interaction with gene expression are not well defined. Here, we demonstrate a complex relationship between control of genome-wide nucleosome positioning and developmental gene expression and its requirement for a Chromodomain-Helicase-DNA binding (CHD) Type III chromatin remodeling protein.

Nucleosome positions can be described by several physical parameters: *occupancy*, the relative enrichment or depletion of nucleosomes at specific DNA sequences; *phase*, the ordered arrangement of nucleosomes relative to a defined genomic feature, e.g., the transcriptional start site (TSS); and *spacing*, the distance between adjacent nucleosomes. A measure of this third parameter is the nucleosome repeat length (NRL), the DNA distance from the center of one nucleosome to the center of the next, which incorporates both the distance between nucleosomes and the contact area of each nucleosome core with DNA. Nucleosome occupancy, phasing, and spacing are under the control of multiprotein chromatin remodeling complexes. Central to each complex is an ATP-dependent DNA translocase from the SWI2/SNF2 family (Becker and Horz 2002; Hargreaves and Crabtree 2011), which has the capacity to change occupancy via nucleosome exchange (Henikoff 2008; Tolstorukov et al. 2013) or change phase and spacing via nucleosome translocation along the DNA backbone (Stockdale et al. 2006; Narlikar et al. 2013; van Bakel et al. 2013).

In yeasts, accumulated evidence indicates interaction between nucleosome positioning and gene regulation (Jiang and Pugh 2009), associated with conformational changes at gene promoters (Yen et al. 2013; Nocetti and Whitehouse 2016) and RNA polymerase progression through gene bodies (Venkatesh and Workman 2015). Nucleosome positioning in the metazoa is more intricate, involving many more remodeling complexes and an increased number of regulatory protein subunits. These remodeling complexes can associate with distinct genetic elements and genomic regions, interact with epigenetic regulators, and act with different functions to activate or repress gene regulation (Zhou et al. 2002; Srinivasan et al. 2008; Yang and Seto 2008; Dorighi and Tamkun 2013; Wu et al. 2014; de Dieuleveult et al. 2016). Although there is in vitro and in vivo evidence to show that chromatin remodeling complexes reposition nucleosomes throughout the genome, it remains unclear to what extent global changes in nucleosome positioning influence the regulation of gene expression (Kadoch and Crabtree 2015). Here, we investigate the relationship between patterns of nucleosome positioning at the whole-genome level and effects on developmentally regulated gene transcription in *Dictyostelium*.

There are multiple SWI2/SNF2-family proteins, including the distinct SWI2/SNF2, ISWI, INO80, and CHD subfamilies (Ryan and Owen-Hughes 2011). These chromatin remodeling complexes are required for development. In *Drosophila,* the remodeler Brahma (Brm) regulates cell fate specification (Tamkun et al. 1992), and its mammalian homologs have roles in T-cell development, stem cell differentiation, and neurodevelopment (Wang et al. 1996; Becker and Horz 2002; Lessard et al. 2007; Hargreaves and Crabtree 2011; Ho et al. 2011). Furthermore, chromatin remodeling proteins have a strong association with human disease, including mental health and cancer (Barnard et al. 2015; Kadoch and Crabtree 2015).

The CHD family has three subtypes (I, II, and III). Subtype III proteins are of particular interest as they regulate multicellular development in animals and *Dictyostelium* and are absent from yeasts (Marfella and Imbalzano 2007; Platt et al. 2013a). Loss-of-function and haploinsufficient mutations of CHD Type III proteins are associated with abnormal multicellular development and embryonic lethality. The *Drosophila* CHD type III protein Kismet (KIS) is required for maintenance of developmental gene activity (Daubresse et al. 1999). In humans, mutation of CHD8 is associated with autism spectrum disorders (ASDs) (De Rubeis et al. 2014; McCarthy et al. 2014; Prontera et al. 2014; Sugathan et al. 2014; Cotney et al. 2015), and haploinsufficient mutations of CHD7 are strongly associated with CHARGE syndrome (Vissers et al. 2004; Lalani et al. 2006; Janssen et al. 2012; Martinez-Quintana et al. 2014) and Kallmann syndrome (Marcos et al. 2014), two severe multisystem disorders. CHD7 can translocate nucleosomes in an in vitro assay (Bouazoune and Kingston 2012), an activity compromised by CHD7 mutations associated with CHARGE syndrome. However, an in vivo role for CHD Type III proteins in nucleosome positioning has not been previously established.

*Dictyostelium discoideum* shares a common evolutionary origin with animals (Baldauf and Doolittle 1997). Upon nutrient depletion, *Dictyostelium* switches from a unicellular growth phase into a program of multicellular development and cell differentiation that utilizes many signaling pathway components, such as phosphotyrosine, as well as alpha- and beta-catenins, generally considered restricted to the metazoa (Kay 1997; Kim et al. 1999; Grimson et al. 2000; Dickinson et al. 2011). We had characterized the three CHD proteins of *Dictyostelium*—ChdA, ChdB, and ChdC—and shown that they are required for expression of discrete subsets of genes and for distinct aspects of growth and development (Platt et al. 2013a). ChdC is an ortholog of metazoan CHD Type III proteins and is absolutely required for progression through multicellular development, conceptually paralleling the congenital defects associated with human CHARGE syndrome. ChdC, therefore, offers the opportunity to investigate CHD Type III remodeling proteins in the context of developmental regulation of nucleosome positioning and gene expression.

Previous nucleosome mapping of *Dictyostelium* chromatin focused on ∼40% of the genome and suggested an organization similar to that of multicellular animals, with little overall developmental change in nucleosome positioning detected at a global level (Chang et al. 2012). Here, we combine nucleosome mapping and gene expression analysis of wild-type (WT) and *chdC*-null mutant cells to probe deeper into the dynamics of *Dictyostelium* nucleosome organization. These results demonstrate a significant role for CHDs in nucleosome positioning and control of developmental gene regulation and offer insight for the function of CHD Type III proteins in metazoan development and in disease mechanisms for related human genetic syndromes.

## Results

### Characterization of *Dictyostelium* nucleosome positioning during growth

We mapped the global distribution of nucleosomes in growing WT *Dictyostelium* chromatin using MNase-seq (Kent et al. 2011; Platt et al. 2013b). Chromatin was digested with micrococcal nuclease (MNase) to create nuclease-resistant DNA ladders with a fragment spectrum of <1 kb and was analyzed by Illumina paired-end DNA sequencing. The resulting aligned paired-read data set was stratified computationally into sizes of 150 ± 30 bp to create a subset of nucleosome-protected DNA fragments. The sequence position of the midpoint of each protected fragment, i.e., the nucleosome “dyad” axis, was calculated and frequency distributions mapped across the entire *Dictyostelium* genome. Variations in frequency values were normalized to the mean values through a −600- to +600-bp window flanking each dyad position (Kent et al. 2011; Zhang and Pugh 2011). Prominent peaks within these distributions indicate the presence of similarly positioned nucleosomes throughout the cell population, and the distance between peaks gives the NRL.

**Figure 1.**
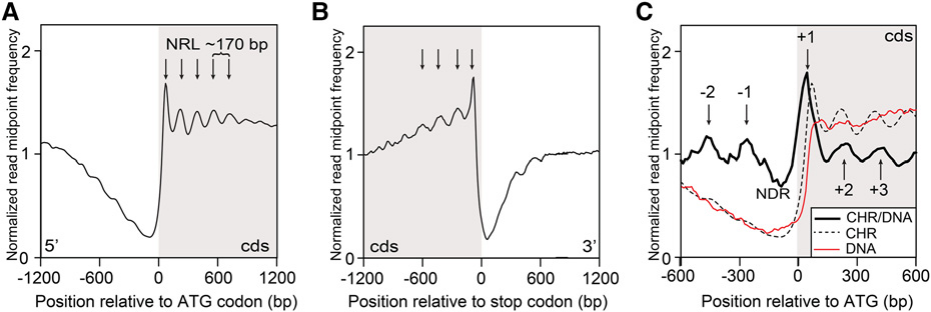
Genome-wide nucleosome positioning in *Dictyostelium*. (*A*) Normalized read midpoint frequency distributions of MNase-protected fragments (nucleosome dyads) of all 12,750 genes in growth-stage WT cells were aligned relative to their ATG codons. Peaks (arrows) correspond to dyad midpoints for globally phased nucleosomes in the 5′ region of intragenic DNA, and distances between mapped read peaks correspond to ∼170 bp NRL. The protein coding DNA sequence (cds) region is shaded. (*B*) Normalized read midpoint frequency distributions of all genes in growth-stage WT cells were aligned relative to their translational termination sites (stop codons). Peaks (arrows) in the mean normalized frequency distribution correspond to globally phased nucleosomes in the 3′ region of intragenic DNA. The protein cds region is shaded. (*C*) Normalized dyad read midpoint frequency distributions for WT chromatin (CHR; dotted line) (see *A*) were adjusted for sequence mappability by dividing with equivalent control data from MNase-digested naked (protein free) WT DNA (DNA; red line) and replotted as the ratio (CHR/DNA; thick black line) within 1.2 kb of flanking chromatin relative to ATG sites of all 12,750 genes. An ∼170-bp nucleosome-depleted (“free”) region (NDR) is centered near the AT-rich regions of *Dictyostelium* TSS. Positioned nucleosomes upstream (+) and downstream (−) to the NDR are indicated by arrows. The protein cds region is shaded.

To view chromatin organization at the whole-genome level, we first aligned all 12,750 protein-coding genes (Basu et al. 2013) relative to the ATG translational start sites (Fig. 1A). As the *Dictyostelium* genome is gene-dense and its genes are mostly small, this alignment visualized >90% of the genome. TSS are not universally annotated in *Dictyostelium* for all genes; nonetheless as the average size of *Dictyostelium* 5′ UTRs (untranslated regions) is very short (<100 bp), the ATG initiation codon serves as a near proxy for the TSS (Basu et al. 2013). Identical nucleosome maps were obtained from independent biological and technical replicates, demonstrating very high reproducibility (Supplemental Fig. S1A; Supplemental Table S1). These analyses based on 12,750 genes are broadly consistent with a previously reported nucleosome organization described for 5468 *Dictyostelium* genes generated by single directional pyrosequencing of isolated mononucleosomes (Chang et al. 2012), indicating that the *Dictyostelium* nucleosome maps are robust across biological repeats, sequence technology platforms, and different WT strains.

Plots showing the nucleosome positions (normalized paired-end read midpoint vs. nucleotide position relative to the ATG codon), calculated as a global average for all genes, show a prominent nucleosome peak 3′ to the ATG initiation codon (Fig. 1A). For growing cells, this nucleosome is followed by regular nucleosome phasing at an average NRL of ∼170 bp for four or more nucleosomes (Supplemental Table S1), a calculated value that is very similar to that estimated (168–170 bp) by electrophoretic mobility of oligo-nucleosomal DNA fragments (Blumberg et al. 1991; Platt et al. 2013b). As the position of the most prominent first nucleosome relative to the ATG varies from gene to gene, we also globally mapped intragenic nucleosome patterns by alignment to each of these +1 nucleosomes (Supplemental Fig. S1B,C); this again showed a highly reproducible, ∼170-bp NRL in growing cells (Supplemental Table S1).

A regular nucleosome pattern indicates that a large component of *Dictyostelium* chromatin is organized with nucleosomes arrayed in phase with respect to the 5′-end of each gene (Fig. 1A), and similar to that seen previously in a more limited gene set (Chang et al. 2012). Comparable arrayed nucleosomes are observed in 3′-regions, when translational stop codons are globally aligned (Fig. 1B), indicative of similar nucleosome organization throughout gene coding regions. To examine how nucleosome positioning varied across the genome, we compared nucleosome patterns of each individual gene by *k*-means cluster analysis. We identified five distinct chromatin clusters with overall patterns that were highly replicated at both biological and technical levels (Supplemental Fig. S2A). Although these clusters differed in their patterns of nucleosome peak heights and phasing relative to the ATG codon, they each possessed an ∼170-bp NRL. We observed no correlation of different clusters with gene length (Supplemental Fig. S2B) or gene expression level measured by RNA-seq (Supplemental Fig. S2C).

In *Dictyostelium*, the intergenic regions and introns are highly (>85%) AT-rich (Eichinger et al. 2005), which can lower read counts in these regions due to compromised sequencing efficiency, reduced ability to unambiguously map some fragments to the genome, and increased MNase cleavage rates. To compensate for lowered read depth of intergenic regions, we compared midpoint frequencies of both MNase-digested naked DNA controls (Fig. 1C) and sonicated naked DNA controls (Supplemental Fig. S3) to our nucleosome dyad frequency data and detected evidence for global nucleosome organization, proximal to the 5′ regions of genes (Fig. 1C). These upstream nucleosomes were separated from the downstream coding region nucleosomes by an ∼170-bp nucleosome-depleted region (NDR) that corresponds to the most highly AT-rich regions of the genome, which are generally found 5′ to *Dictyostelium* protein coding sequences (Segal and Widom 2009; Zhang et al. 2011; Chang et al. 2012). Similar NDR promoter regions have been reported in other species, such as yeast, fly, mouse, and human cells (Lee et al. 2007; Mavrich et al. 2008; West et al. 2014).

### Gene-specific nucleosome remodeling during development

**Figure 2.**
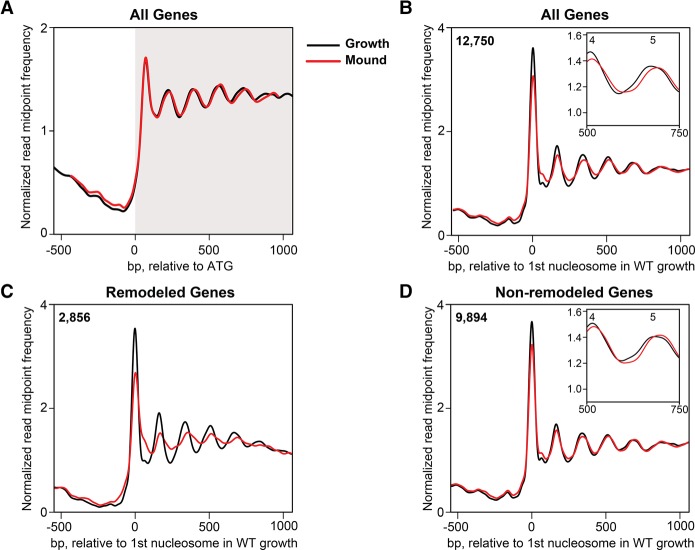
Nucleosomes are remodeled in a subset of genes during WT development. (*A*) Normalized read midpoint frequencies in growing-stage (black) and loose-mound–stage (red) WT cells for all 12,750 genes aligned to their ATG sites. (*B*) Normalized read midpoint frequencies in growing-stage (black) and loose-mound–stage (red) WT cells for all 12,750 genes aligned to the midpoint of the first defined nucleosome in growing WT cells. (*Inset*) Enlarged view of nucleosomes 4 and 5 to show the +3-bp NRL shift from growth to development. (*C*) Normalized read midpoint in growing-stage (black) and loose-mound–stage (red) WT cells for the 2856 developmentally remodeled genes aligned to the midpoint of the first defined nucleosome in growing WT cells. (*D*) Normalized read midpoint frequencies in growing-stage (black) and loose-mound–stage (red) WT cells for the 9894 non-remodeled genes aligned to the midpoint of the first defined nucleosome in growing WT cells. (*Inset*) Enlarged view of nucleosomes 4 and 5 to show the increased NRL from growth to development.

Global nucleosome patterns from *Dictyostelium* were sampled at the multicellular, loose-mound stage of development and found to be largely similar to that of growing cells (Fig. 2A). When aligned to the ATG codon, the read midpoint frequency plot for all genes indicated similarly positioned nucleosomes and downstream phasing in developed and growth-stage cells (Fig. 2A). *k*-means cluster analysis also showed very similar patterns at both stages (Supplemental Fig. S4A). To directly compare the nucleosome organization of growth and loose-mound cells, we aligned both sets to the +1 nucleosome position for WT growth chromatin (Fig. 2B). Such analyses allow comparison of intragenic nucleosome patterns between different developmental stages (and mutant strain types; see below), irrespective of variability of nucleosome positioning relative to individual ATGs and the underlying backbone sequence. The global nucleosome patterns of growing and developed cells were very similar overall (Fig. 2A,B). We did, however, observe a minor, but reproducible, increase of ∼3 bp in the global average NRL in loose-mound–stage cells, ∼173 bp compared to the ∼170 bp seen in replicate data sets of growing cells (Fig. 2A,B; Supplemental Fig. S5; Supplemental Table S1); an increased NRL was previously also observed at an earlier stage of development by Chang et al. (2012).

To investigate whether more substantial changes in nucleosome patterning were masked by global averaging, we queried each of the 12,750 genes for three or more differences in nucleosome position and/or peak-height between the growth and loose-mound cells. This identified 2856 genes with a different nucleosome organization between the growing and developing cell populations. When the nucleosome pattern of this gene set was directly compared, we observed substantial developmental-dependent differences with significant loss of nucleosome organization compared to growth (Fig. 2C; Supplemental Fig. S4B); we termed these genes as “remodeled.” The remaining non-remodeled 9894 genes were nearly indistinguishable from growth-stage chromatin, except for the increased NRL globally characteristic of the loose-mound–stage cells (Fig. 2D; Supplemental Fig. S4C). These results indicate that during mound-stage development, ∼20% of the *Dictyostelium* genome undergoes significant nucleosome remodeling, while nucleosome positions through most of the genome remains largely unchanged. Remodeling was observed across all five chromatin cluster groups (Supplemental Fig. S4B).

**Figure 3.**
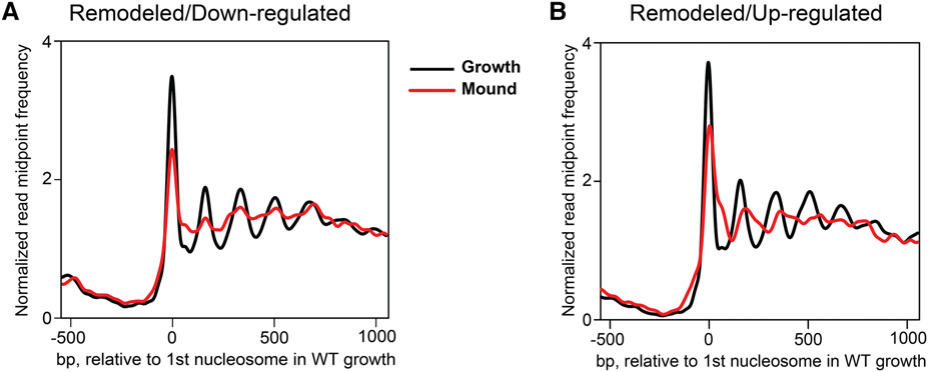
Nucleosome remodeling occurs in genes whose expression is down-regulated or up-regulated during development. Normalized read midpoint frequencies in growing-stage (black) and loose-mound–stage (red) WT cells for developmentally remodeled genes aligned to the midpoint of the first defined nucleosome in growing WT cells. (*A*) Remodeled genes whose expression is down-regulated from growth to the loose-mound stage (see Supplemental Fig. S6). (*B*) Remodeled genes whose expression is up-regulated from growth to the loose-mound stage (see Supplemental Fig. S6).

To relate these developmentally regulated chromatin changes to differences in gene expression, we analyzed transcriptome profiles generated by RNA-seq in the same cell preparations used for our MNase-seq analysis. We found that about 7000 (∼55% of all) genes exhibit expression differences when growth and loose-mound stages are compared (Supplemental Fig. S6). Approximately half of these genes are up-regulated and half are down-regulated (Loomis and Shaulsky 2011), reflecting highly dynamic transcriptional changes throughout the genome during development (Supplemental Fig. S6). Although the developmentally remodeled genes represent only ∼20% (∼2850) of the total genome, they are significantly enriched for genes (1700) that are differentially expressed at the loose-mound stage (*P* < 4 × 10^−7^), as per hypergeometric distribution. Developmentally regulated remodeling was observed equally in genes whose expression was either elevated or suppressed during development (Fig. 3).

These data indicate a significant relationship between nucleosome positioning and developmentally regulated gene expression. Still, it should be noted that many genes exhibit altered gene expression during development without an accompanying change in nucleosome organization, indicating that developmental changes in transcriptional activity per se is not universally reflective of substantial nucleosome repositioning or displacement.

### Altered gene-specific, nucleosome spacing in *Dictyostelium* lacking ChdC

ChdC is one of three CHD chromatin remodeling orthologs in *Dictyostelium* and is a member of the CHD Type III protein subfamily. Its expression peaks at ∼8–12 h of development as cells enter the loose-mound stage, and has a major developmental role, as *chdC*-null cells undergo developmental arrest at this stage due to substantial misregulation (∼50%) of genes required for aggregation or cell fate organization pathways (Platt et al. 2013a). The *chdC*-null mutants, therefore, provide a genetic probe for investigating the developmental role of nucleosome positioning and offer a paradigm for investigation of the in vivo role of CHD Type III proteins in developmental regulation.

**Figure 4.**
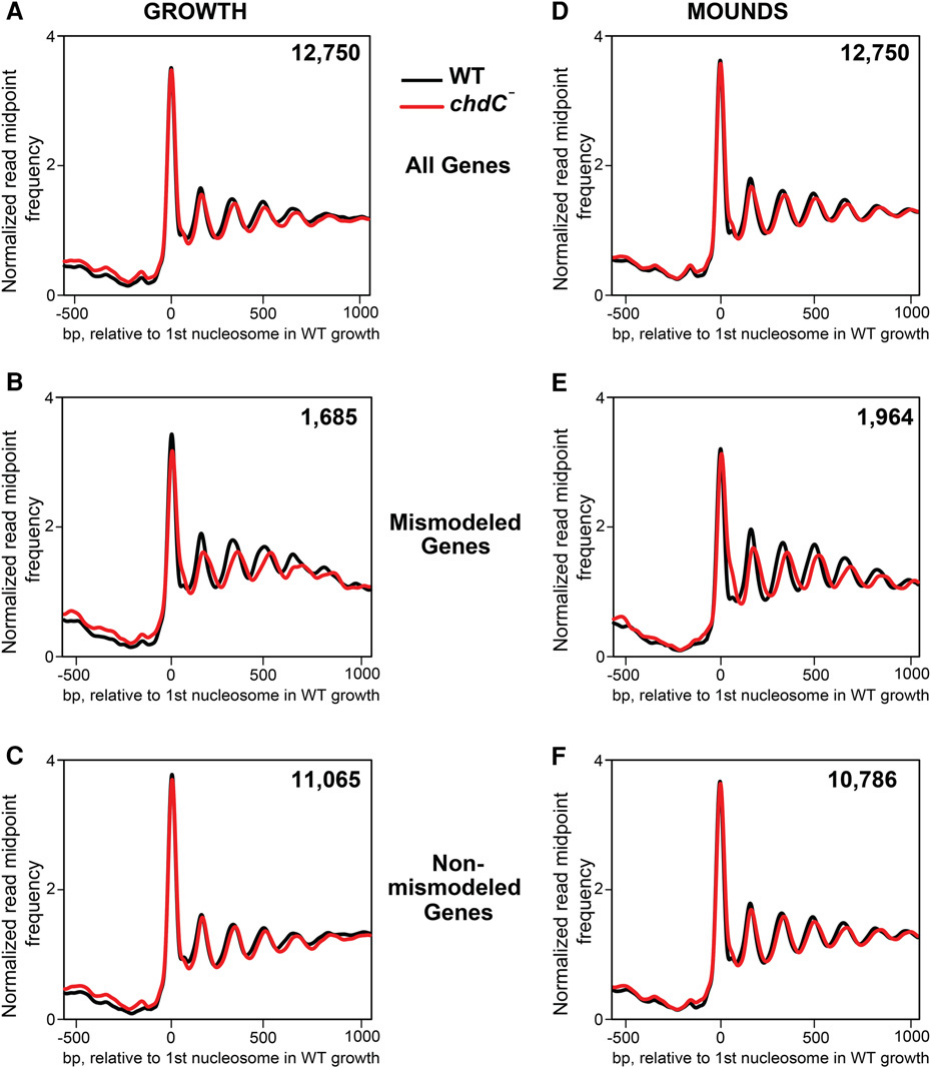
Nucleosomes are mismodeled in a subset of genes during growth and development of *chdC*-null cells. (*A*) Normalized read midpoint frequencies in WT (black) and *chdC*-null (red) growing cells for all 12,750 genes aligned to the midpoint of the first defined nucleosome in WT growing cells. (*B*) Normalized read midpoint frequencies in WT (black) and *chdC*-null (red) growing cells for the 1685 mismodeled genes in growing *chdC*-null cells aligned to the midpoint of the first defined nucleosome in WT growing cells. (*C*) Normalized read midpoint frequencies in WT (black) and *chdC*-null (red) growing cells for the 11,065 non-mismodeled genes in growing *chdC*-null cells aligned to the midpoint of the first defined nucleosome in WT growing cells. (*D*) Normalized read midpoint frequencies in WT (black) and *chdC*-null (red) loose-mound–stage cells for all 12,750 genes aligned to the midpoint of the first defined nucleosome in WT growing cells. (*E*) Normalized read midpoint frequencies in WT (black) and *chdC*-null (red) loose-mound–stage cells for the 1964 mismodeled genes in mound-stage *chdC*-null cells aligned to the midpoint of the first defined nucleosome in WT growing cells. (*F*) Normalized read midpoint frequencies in WT (black) and *chdC*-null (red) loose-mound–stage cells for the 10,786 non-mismodeled genes in loose-mound–stage *chdC*-null cells aligned to the midpoint of the first defined nucleosome in WT growing cells.

We compared equivalent nucleosome maps generated from *chdC*-null cells to those of WT, examining both growing and developing cells. To control for temporal differences between strains, we used cells that had been developed to the same morphological structure, the loose-mound stage (10 h for WT and 12 h for the *chdC*-null mutant). At both the growth and loose-mound stages, our analyses showed broadly similar global patterns of nucleosome positioning between *chdC*-null and WT cells, when aligned to either the ATG (Supplemental Fig. S7A) or the +1 nucleosome in WT cells (Fig. 4A,D). However, *chdC*-null cells showed an NRL increase of 5 bp for growth-stage and 3 bp for loose-mound–stage cells compared with equivalent WT cells (Supplemental Tables S2, S3).

As per the previous comparison between WT growth and development, we searched for variation in nucleosome patterns and identified 1685 genes in growing cells and 1964 genes at the loose-mound stage that had reproducible differences in chromatin organization in biological and technical replicates between WT and *chdC*-null cells (Fig. 4B,E). By these criteria, ∼15% of all genes in *chdC*-null cells at either stage have differences in nucleosome patterns compared with WT; we termed this gene set as “mismodeled.”

We compared the nucleosome maps of the mismodeled genes in *chdC* nulls to the same genes in WT cells based on alignment to the midpoint of the +1 nucleosome from WT (Fig. 4B,E) or to the ATG (Supplemental Fig. S7B). Nucleosome arrays within all genes, both mismodeled and non-mismodeled, were well phased at the growth and loose-mound stages. Significantly, however, the average NRL for the mismodeled genes was increased to ∼181 bp in the *chdC* nulls compared with ∼169 bp for the same gene set in WT (Supplemental Tables S2, S3). The same 181-bp NRL was observed in both the growth and loose-mound stage, suggesting that nucleosome spacing of genes affected by ChdC expands to a maximum length regardless of developmental state (Supplemental Fig. S8). In contrast, only small differences in nucleosome spacing were seen in genes that were not assigned as mismodeled (Fig. 4C,F).

**Figure 5.**
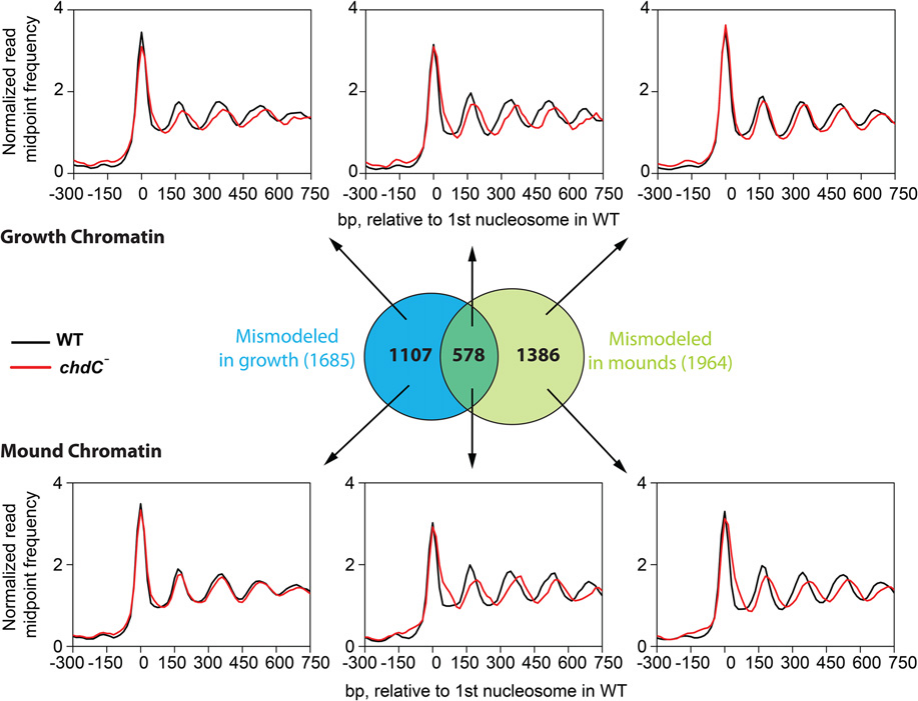
Genes are mismodeled at multiple stages of the *chdC*-null life cycle. Venn diagram indicates significant (*P* < 2 × 10^−16^) overlap among genes that are mismodeled in both growing-stage and loose-mound–stage *chdC*-null cells; *P*-value was calculated using the hypergeometric test. (*Top*) When the different mismodeled gene sets are analyzed for paired read midpoint frequency distributions in growing WT (black) or *chdC*-null (red) cells, only genes that are mismodeled in growing cells exhibit an increased NRL in growing cells; genes that are characterized as only mismodeled in loose-mound–stage cells do not exhibit this increased NRL in growing cells. (*Bottom*) When the different mismodeled gene sets are analyzed for paired read midpoint frequency distributions in loose-mound–stage WT (black) or *chdC*-null (red) cells, only genes that are mismodeled in loose-mound–stage cells exhibit an increased NRL in loose-mound–stage cells; genes that are characterized as only mismodeled in growing cells do not exhibit this increased NRL in loose-mound–stage cells.

Given that similar numbers of genes are mismodeled in growing-stage and loose-mound–stage *chdC*-null cells, we examined whether these were the same or distinct gene sets. Although there is a statistically significant overlap (∼33%; *P* < 2 × 10^−16^) between the two gene populations (Fig. 5), most genes in each set exhibit mismodeling only during growth or only during development. We therefore conclude that ChdC is active throughout growth and early multicellular development, regulating distinct loci at different stages.

**Figure 6.**
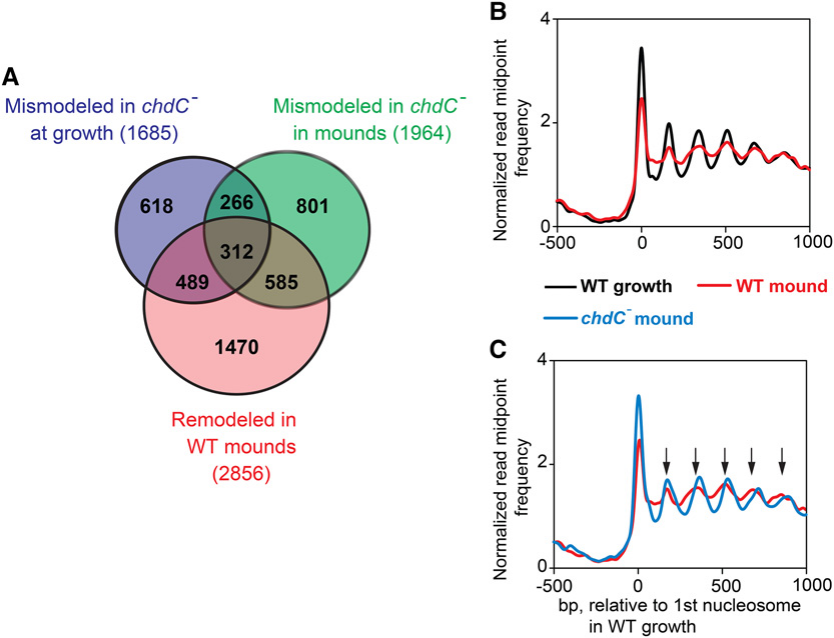
Genes that are remodeled during WT development but mismodeled in *chdC* nulls. (*A*) Venn diagram compares the gene sets mismodeled during growth and development of *chdC*-null cells to the gene set that is remodeled during WT development. (*B*) Normalized read midpoint frequencies of genes classified as both remodeled and mismodeled were determined for WT cells at growth stage (black) or the loose-mound stage (red) aligned to the midpoint of the first defined nucleosome in growing WT cells. (*C*) Normalized read midpoint frequencies of genes classified as both remodeled and mismodeled were determined for loose-mound stage of WT (red) or *chdC*-null mutant (blue) cells aligned to the midpoint of the first defined nucleosome in growing WT cells. Black arrows indicate positions of nucleosomes of WT cells during growth relative to the first nucleosome.

Most significantly, ∼50% of genes that are remodeled in WT cells during development showed a major NRL increase in *chdC*-null mutant cells (Fig. 6A). To directly compare these effects, we aligned the genes that are both remodeled in WT and mismodeled in *chdC*-null cells at the loose-mound stage and observed dramatic differences in chromatin organization (Fig. 6B,C). In addition to increased nucleosome spacing, nucleosome phasing of these genes in *chdC*-null mutants is distinctly more structurally organized compared with WT loose mounds (Fig. 6C), suggesting that, for some genes, ChdC-dependent positioning effects may extend beyond nucleosome spacing. Together these data demonstrate a substantial requirement for ChdC for nucleosome positioning during multicellular development.

### A complex relationship between nucleosome spacing and gene expression in *chdC* mutants

Previously, we had shown that loss of *chdC* caused an extensive misexpression of genes during growth and the cAMP pulse-regulated aggregation stage of development (Platt et al. 2013a). To investigate more directly the potential relationship between altered gene expression and mismodeled chromatin organization in the absence of ChdC, we made a new comparative RNA-seq analysis (Supplemental Fig. S9) using the same growth-stage and loose-mound–stage cell preparations analyzed in our MNase-seq experiments (see Fig. 4). In growing cells, 939 genes were up-regulated by greater than twofold (*P* < 0.05) and 667 genes were down-regulated by greater than twofold (*P* < 0.05) in *chdC* nulls compared with the WT (Supplemental Fig. S9), corresponding to ∼13% of all *Dictyostelium* genes. A similar trend was also seen in developed cells with 2288 genes up-regulated by greater than twofold in *chdC* nulls (*P* < 0.05) and 2022 genes down-regulated by greater than twofold (*P* < 0.05), representing an even greater portion of genes (∼35%) with altered expression patterns (Supplemental Fig. S9).

**Figure 7.**
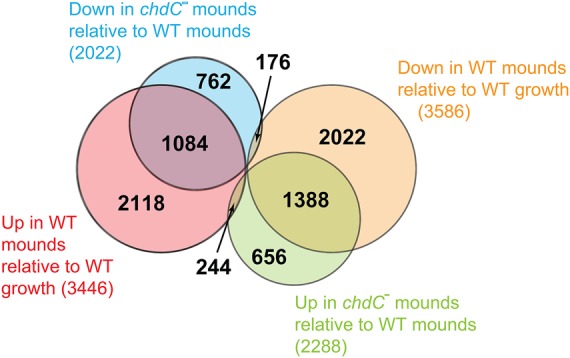
ChdC is required for regulated gene expressed during WT development. Euler diagram compares the gene sets that are misexpressed at the loose-mound stage of *chdC*-null cells to the gene sets that are developmentally regulated during WT loose-mound formation.

The RNA-seq analysis of loose-mound–stage cell differentiation extends the gene expression differences seen at the aggregation stage and largely explains the phenotypic defects of *chdC* nulls during multicellular differentiation (Platt et al. 2013a). For example, at the loose-mound stage, >54% of genes annotated as either prespore or prestalk specific had greater than twofold reduced expression in *chdC*-null cells compared with the WT. A particularly significant underexpressed mRNA is *car2,* which encodes a cAMP receptor required for progression and coordination of multicellular development beyond the mound stage (Saxe et al. 1993). In toto, >50% of genes that are underexpressed in *chdC*-null loose mounds are normally up-regulated at this stage of WT development, and >60% of genes that are overexpressed in *chdC*-null loose mounds are normally down-regulated during WT development (Fig. 7). Collectively, these RNA-seq data (Fig. 7; Supplemental Fig. S9) demonstrate a major deficit in developmental gene expression in *chdC*-null cells that can be directly linked to mound-stage mutant phenotypes via transcriptional changes.

Although these data indicate that ChdC is required for developmental regulation of both nucleosome positioning (Fig. 4) and gene expression (Fig. 7; Supplemental Fig. S9), we wished to determine if these processes were functionally coupled. We examined the correlation of gene mismodeling and misexpression in *chdC*-null cells during growth and development in comparison to the WT. In growing cells, 572 of 1685 mismodeled genes also had aberrant gene expression, a significant enrichment (*P* < 10^−15^). A similar relationship is observed at the loose-mound stage, where 711 of 1964 genes are both mismodeled and misexpressed (*P* < 0.003); however, this developmental analysis is confounded by the large number of genes (2700) that are misregulated, but not mismodeled, at the mound stage. *Dictyostelium* development is comprised of a series of dependent steps (Loomis et al. 1976) that can each have consequences for gene expression at later development stages. Many compounding gene expression changes observed at the mound stage of ChdC-null mutants may, therefore, arise as indirect consequences of previous developmental changes. To better address this, we examined only those genes that are both developmentally remodeled and developmentally regulated in WT cells. When this restricted gene set was compared for variance to *chdC* nulls, we observe a remarkably high correlation, (*P* < 10^−13^), demonstrating a strong statistically significant association between misregulation of nucleosome position and gene expression in *chdC*-null cells.

## Discussion

We have demonstrated developmentally regulated repositioning of nucleosomes in *Dictyostelium* for a subset of genes during the transition from growth to the multicellular stage of development. We showed that 50% of these genes require ChdC for chromatin remodeling, and more than 1200 genes exhibit both mismodeled nucleosomes and aberrant gene expression at some stage in *chdC*-null mutant cells. Our statistical analysis indicates that these effects do not behave as independent variables, providing evidence for functional coupling between ChdC-mediated nucleosome positioning and gene regulation.

We find that during WT growth, *Dictyostelium* genes possess a general nucleosome pattern of a prominent +1 nucleosome that lies 3′ to a presumptive TSS and is followed by a series of phased nucleosomes with an average NRL of ∼170 bp. Globally, we define an NDR at −115 bp relative to the ATG, a first coding nucleosome at +60 bp relative to the ATG, and an average intergenic NRL of ∼170 bp. In addition, by adjusting paired-end read midpoint frequencies for mappability across the very AT-rich *Dictyostelium* promoter elements, we provide evidence for upstream nucleosome phasing with a first upstream nucleosome at −250 bp relative to the ATG. This overall 5′ organization, including the phased, −1 upstream nucleosome, very closely parallels that previously suggested for *Dictyostelium* (Chang et al. 2012) using a wholly different approach and alignment to TSS, where available.

Most genes do not exhibit a major global change in nucleosome pattern as cells develop to the loose-mound stage, with the exception of a small increase in average NRL; a developmental increase in NRL was similarly observed by Chang et al. (2012). The functional significance of this increase is unknown, but as a complete turn of the DNA helix occurs every 10.5 bp, small differences in nucleosome spacing may lead to substantial rotational rearrangements of nucleosome packing and chromatin organization (Correll et al. 2012; Grigoryev 2012). An average 3- to 5-bp NRL increase could indicate substantial structural changes of chromatin organization throughout the nucleus.

Importantly, detailed analysis of nucleosome patterns for individual genes identified a subset of genes with substantial changes in nucleosome pattern and associated changes in gene expression as cells develop to the loose-mound stage. Chromatin changes were represented in genes that were developmentally up-regulated or down-regulated, indicating that altered chromatin structure is neither wholly activating nor repressive. Approximately 50% of genes that are remodeled during WT *Dictyostelium* development are also mismodeled in cells lacking ChdC. As the majority of mismodeled genes are different between growth and loose-mound stage, ChdC is likely to be active throughout development with its gene targets being respecified as development proceeds. Significantly, genes that are misexpressed in *chdC*-null cells during mound formation account for ∼50% of the genes whose transcription is specifically regulated at the mound stage of WT development. These observations indicate that ChdC is a major regulator of both nucleosome positioning and gene expression as cells enter the mound-stage development.

Our data provide the first in vivo study for action of a CHD Type III protein on chromatin remodeling. In vitro studies had previously shown that ISWI and CHD proteins exhibit an ATP-dependent nucleosome sliding activity on artificial DNA templates (Hamiche et al. 1999; Langst et al. 1999; Stockdale et al. 2006; Bouazoune and Kingston 2012). More recently, it was shown that CHD7 proteins containing mutations that are associated with the human developmental disorder CHARGE syndrome have highly impaired remodeling activity in vitro compared with WT CHD7 (Bouazoune and Kingston 2012). Our in vivo evidence is supportive of the previous in vitro biochemical studies, showing that cells lacking ChdC have a very specific deficit resulting in an expanded NRL.

On a gene-by-gene basis, however, the remaining ∼50% of genes that are mismodeled in *chdC*-null cells have no detectable changes in steady-state RNA levels. Experimentally, RNA-seq may be insufficiently sensitive to detect subtle changes in gene transcription. *Dictyostelium* transcription has been shown to occur in brief bursts that can be measured in individual cells (Corrigan and Chubb 2014). Persistence and decay of these bursts occur on a faster timescale than the developmental time course used in our global analysis and may not be reflected in an overall population measure. Monitoring transcriptional bursts or other behaviors may offer a more sensitive sensor of nucleosome effects on gene expression. Gene expression variations that result from limited asynchrony or are restricted to minor subpopulations of cells that differentiate during mound formation can also add complexity. Still, these results indicate that associations can be measured at the global level, although an altered NRL may not be sufficient to predict misregulation of gene expression at the individual gene level.

*Dictyostelium* possesses multiple spacing chromatin remodelers, three CHD proteins (A, B, and C) and a single ISWI protein, that are predicted to translocate nucleosomes along the DNA backbone (Hamiche et al. 1999; Langst et al. 1999; Stockdale et al. 2006; Bouazoune and Kingston 2012). In mammalian stem cells, these complexes are likely to work in concert but act upon different aspects of transcription (de Dieuleveult et al. 2016). If the combinatorial effect of these different spacing remodelers varies among genes, only a fraction of genes may be strongly sensitive to loss of *chdC*. Furthermore, nucleosome positioning may not determine transcriptional on or off state but, instead, may modulate the degree of transcriptional stimulation or repression. CHDs may act globally to allow a permissive chromatin organization, but local transcription factors may ultimately determine gene activation. Finally, additional proteins in the multiple CHD-containing protein complexes may function in parallel and contribute to gene regulation. In this context, KIS, the *Drosophila* CHD Type III, increases gene activation via H3K36me2/3 and decreases repression via loss of H3K27me3 (Srinivasan et al. 2008; Schnetz et al. 2009; Dorighi and Tamkun 2013). *Dictyostelium* similarly possesses both activating and suppressive histone modifications (Chubb et al. 2006; Kaller et al. 2006), and ChdC may mediate changes in histone modification and transcriptional activity for some gene targets.

To conclude, our analysis uses the small *Dictyostelium* genome to provide a global description of developmentally regulated nucleosome positioning. We demonstrate that the CHD Type III chromatin remodeler ChdC carries out a specific structural role by controlling nucleosome spacing and transcriptional regulation of a limited gene set. These data demonstrate a complex relationship between nucleosome positioning and gene expression that can be detected at a genome-wide scale but is, however, not wholly sufficient to define transcription at a local gene basis. Current evidence suggests a similar complexity in the interaction between chromatin structure and gene regulation in animal development and human genetic diseases. *Dictyostelium* offers an experimentally tractable organism to probe complexities of multicellular development using genetic, molecular, and genomic tools.

## Methods

### *Dictyostelium* strains and development

*Dictyostelium* Ax2 (WT) and *chdC*-null cells (Platt et al. 2013a) were grown axenically in HL5 medium at 20°C; *chdC*-null cells were maintained in 10 µg/mL blasticidin S. For development, growing cells in log phase (1–3 × 10^6^ cells/mL) were washed twice in KK2 buffer (15 mM KH_2_PO_4_, 3 mM K_2_HPO_4_) and developed on 0.45-µm nitrocellulose filters to the identical loose-mound morphologic stage, 10 h for WT and 12 h for *chdC* nulls.

### Chromatin isolation, MNase digestion, and paired-end DNA sequencing

MNase-seq was as previously described (Kent et al. 2011; Platt et al. 2013b). Briefly, 1 × 10^9^ cells were washed in 100 mM sorbitol and resuspended in 400 µL digestion buffer (100 mM sorbitol, 50 mM NaCl, 10 mM Tris-HCl at pH 7.5, 5 mM MgCl_2_, 1 mM CaCl_2_, 1 mM 2-mercaptoethanol, 0.5 mM spermidine, 0.1% Nonidet P40) and transferred to a 1.5-mL microcentrifuge tube containing MNase (USB/Affymetrix) at a final concentration of 800 U/mL; incubation was at 37°C for 2 min. Digestion was stopped by addition of 40 µL stop buffer (5% SDS, 250 mM EDTA at pH 8.4), followed by phenol/chloroform extraction of DNA. RNase A treatment was for 30 min at 37°C. The DNA was re-extracted with phenol/chloroform and precipitated with sodium acetate and 100% ethanol. Three independent MNase digests for each sample were carried out, pooled, and separated on a 1.5% agarose gel. The 50- to 1000-bp gel region was extracted and libraries prepared with the Illumina paired-end kit. Libraries were 76-bp paired-end sequenced on the Illumina HiSeq 2000 at a relatively low density of about 350,000 clusters/mm^2^. Two biologically independent MNase-seq libraries were analyzed for each developmental stage of WT and *chdC*-null mutants and compared (see Supplemental Figs. S1A,B). All figures show data from biologically replicated samples; although they match so closely, they are generally indistinguishable. Purified WT DNA controls were fragmented by MNase digestion or sonication to a similar size range and sequence-processed in parallel.

### Data analysis for nucleosome positioning

The paired-end reads, clipped to 36 bp, were aligned to the *Dictyostelium* genome (see Basu et al. 2013) using Bowtie v0.12.7 (Langmead et al. 2009), and sizes of MNase-protected species were inferred from the end-to-end distances of the paired sequence tags (Kent et al. 2011). For nucleosome mapping, read-pairs were selected at a SAM format ISIZE of 150 bp (±30 bp) and filtered using a simple heuristic peak marker that reports the bins corresponding to nucleosome read midpoint frequency maxima above a noise threshold (Gal et al. 2015). Frequency distributions of these read midpoints (representing the putative nucleosome dyad positions) were plotted relative to the *Dictyostelium* genome in 1-bp bins using read midpoint frequency distributions normalized to the average frequency value within the sequence window and rendered with the Integrated Genome Browser (IGB) (Nicol et al. 2009). The +1 nucleosome was defined as the first nucleosome to appear 5′ to the ATG within each gene. To calculate NRL, the cumulative distances relative to the +1 nucleosome were plotted for each of the first five nucleosomes (based on maximum peak height values) of all gene coding regions (Supplemental Figs. S5, S8) and used to fit linear regression equations from which the average NRL was calculated. Nucleosome patterns were clustered with Cluster 3.0, using Euclidean distance as the similarity metric, *k* = 5, and tested by multiple trials and alternative similarity metrics (de Hoon et al. 2004). Heatmaps were rendered in Java TreeView (Saldanha 2004). For determining differences in nucleosome positions, nucleosome dyad frequency data were smoothed to minimize noise using an Epanechnikov kernel density estimate (Kernel Estimation-0.05 with bandwidth = 30) (Gal et al. 2015). Nonmatching peak-summits, defined as variances of greater than twofold in peak frequency or >10-bp mismatch in peak summit position, were determined in comparison of two data sets (e.g., growth/development, WT/*chdC* nulls). Differentially modeled (remodeled for WT or mismodeled for *chdC* nulls) genes were identified by cross-comparison of paired read midpoint position and peak value date, and selected only if they reproducibly contained three nonmatching nucleosomes (differing in either nucleosome position or peak height) within 1000 bp 3′ to the ATG start site, for two independent biological replicates. The significance of the overlap between gene lists was determined using the hypergeometric distribution (Fury et al. 2006).

### RNA extraction and RNA sequencing

RNA was isolated from WT and *chdC*-null cells strains using TRIzol (Invitrogen) and following the manufacturer's instructions. RNA quality was confirmed using the Agilent 2100 Bioanlyzer, and 5 µg of total RNA was used for poly(A) enrichment and Illumina library preparation, following the manufacturer's instructions. Libraries were 50-bp single-end sequenced on the Illumina HiSeq 2000. The resulting sequence reads were aligned to the *Dictyostelium* genome with TopHat (version 1.3.0) (Trapnell et al. 2009) using the gene models from dictyBase (http://dictybase.org/). FPKM (fragments per kilobase of exon per million reads) and differential expression were calculated using HTSeq and DESeq (Anders and Huber 2010). Plots were drawn in R (R Core Team 2016). Two biologically independent samples were analyzed for each stage of WT and *chdC*-null mutant cells (Supplemental Fig. S10).

## Data access

Raw sequencing data from this study have been submitted to the NCBI Gene Expression Omnibus (GEO; https://www.ncbi.nlm.nih.gov/geo/) under accession number GSE70122. Raw RNA-seq data from this study have been submitted to GEO under accession number GSE70141.

## Supplementary Material

Supplemental Material
