# Supplementary material for: Regulation of nucleosome positioning by a CHD Type III chromatin remodeler and its relationship to developmental gene expression in Dictyostelium
Source: Genome Res. 2017 Apr;27(4):591–600. doi: 10.1101/gr.216309.116 (PMC5378177; doi:10.1101/gr.216309.116)
Supplement: Supplemental Material [file supp_27_4_591__index.html]

Regulation of nucleosome positioning by a CHD Type III chromatin remodeler and its relationship to developmental gene expression in Dictyostelium — Supplemental Material 

# Regulation of nucleosome positioning by a CHD Type III chromatin remodeler and its relationship to developmental gene expression in *Dictyostelium*

## Supplemental Material

- Supplemental\_Material.pdf
