# Supplementary material for: Regulation of nucleosome positioning by a CHD Type III chromatin remodeler and its relationship to developmental gene expression in Dictyostelium
Source: Genome Res. 2017 Apr;27(4):591–600. doi: 10.1101/gr.216309.116 (PMC5378177; doi:10.1101/gr.216309.116)
Supplement: Supplemental Material [file supp_gr.216309.116_Supplemental_Material.pdf]

SUPPLEMENTAL INFORMATION

Supplemental Figures.

		Page
Figure S1	Nucleosome positioning data are highly repeatable	2
Figure S2	Cluster analysis of nucleosome positioning data.	3
Figure S3	Comparison of MNase-digested chromatin to sonicated naked DNA.	4
Figure S4	Nucleosomes are remodeled in a subset of genes during WT development.	5
Figure S5	Global nucleosome repeat length (NRL) differs in WT between growth and development.	6
Figure S6	Developmentally regulated gene expression during WT development.	7
Figure S7	Nucleosomes are mismodeled in a subset of genes during growth of *chdC*-null cells.	8
Figure S8	Nucleosome repeat length (NRL) is lengthened in a subset of genes in *chdC*-null cells during growth and development.	9
Figure S9	The *chdC*-null phenotype correlates with large changes in gene expression during growth and development.	10
Figure S10	Reproducibility of RNA-seq data for WT and *chdC*-null cells	11

Supplemental Tables

Table S1	Nucleosome Spacer Differences: Loose Mound/Growth, WT Cells	12
Table S2	Nucleosome Spacer Differences: *chdC*$^{-}$/WT Cells, Growth	13
Table S3	Nucleosome Spacer Differences: *chdC*$^{-}$/WT Cells, LM	14

Figure S1. Nucleosome positioning data are highly repeatable.

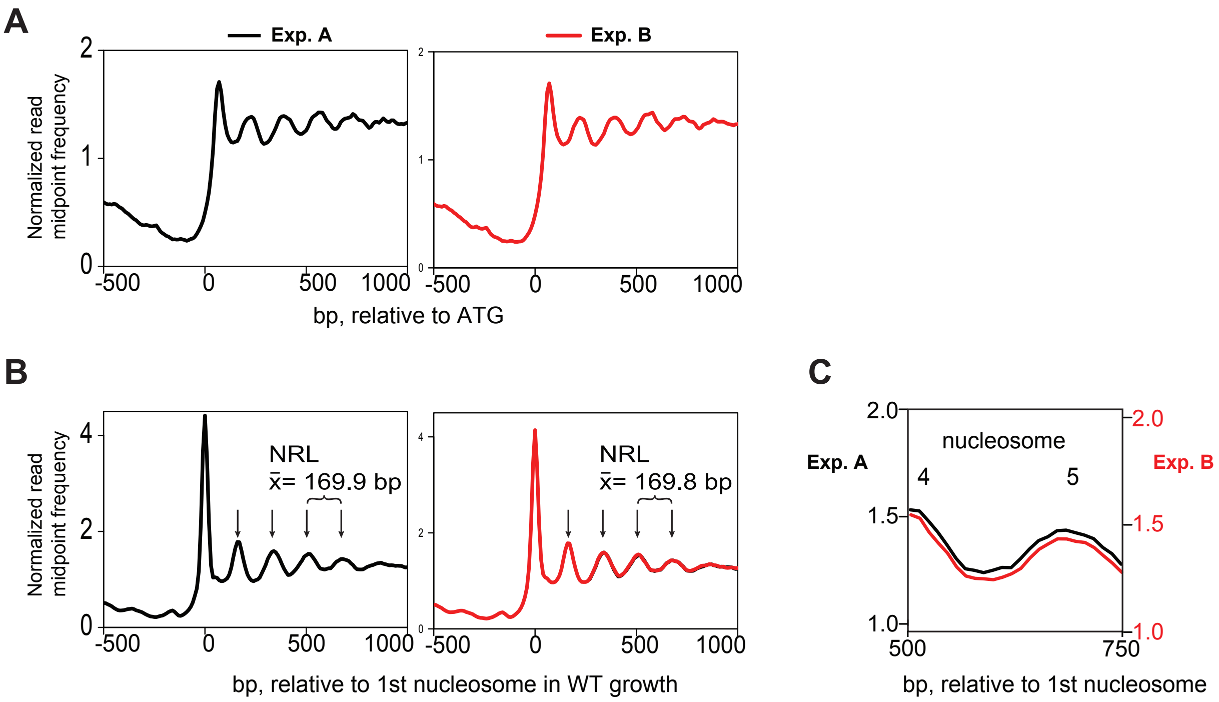

Figure S1. Nucleosome positioning data are highly repeatable.

A. Normalized read midpoint frequency distributions of MNase protected fragments (nucleosome dyads) of all 12,750 genes in growth stage WT cells, aligned to their ATG sites, for two biological and technical independent experiments (Exp. A, black; Exp. B red).

B. Normalized read midpoint frequency distributions of MNase protected fragments (nucleosome dyads) of all 12,750 genes in growth stage WT cells, aligned to the midpoint of the first defined (+1) nucleosome, for two biological and technical independent experiments (Exp. A, black; Exp. B red).

C. Enlarged view of Fig. S1B nucleosome positions 4 and 5 for Exp. A (black) and Exp. B (red). The plot for independent replicate B is offset to allow clear visualization of the overlapping lines.

Figure S2. Cluster analysis of nucleosome positioning data.

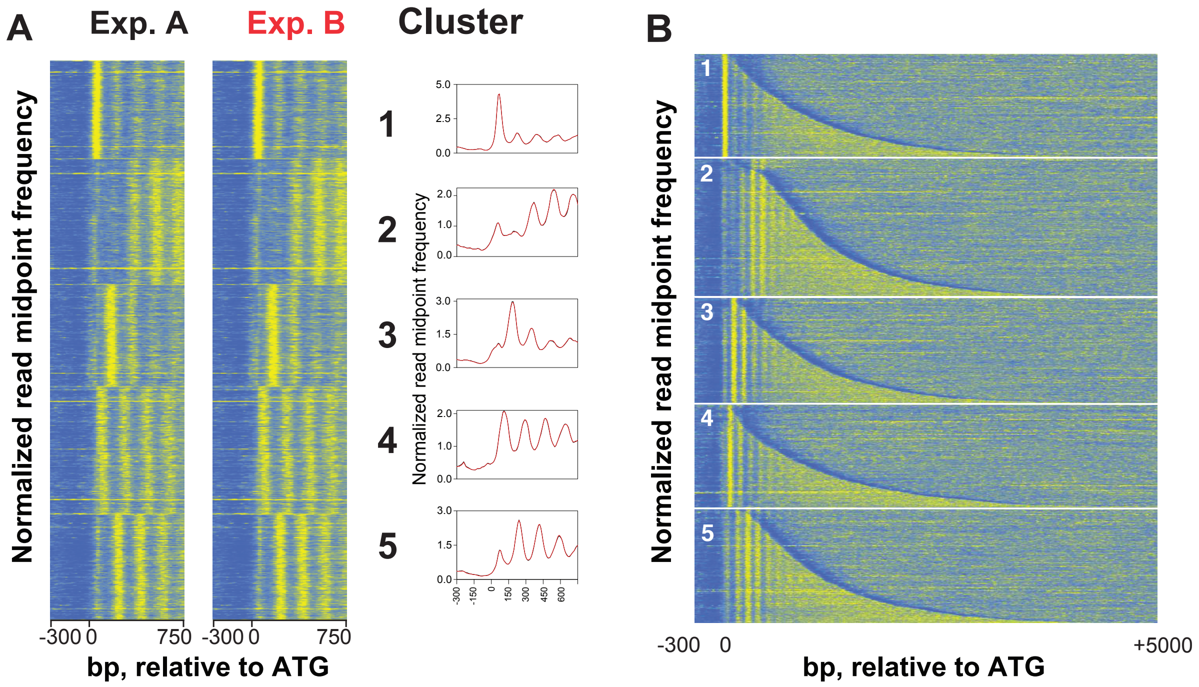

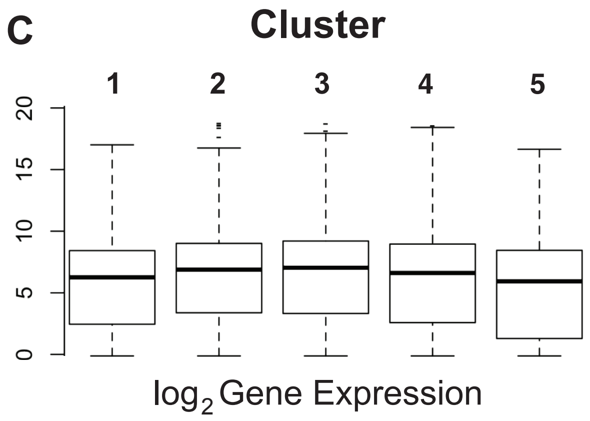

Figure S2. Cluster analysis of nucleosome positioning data.

A. The nucleosome maps of all 12,750 genes aligned by their ATG sites were k-means clustered into 5 groups and displayed by heat map distribution for two biological and technical independent experiments. Right panels show nucleosome frequency plot (read depth of dyad positions) for each cluster group of each experimental repeat (black or red lines). Gene orders were determined from experimental A (back line).

B. The nucleosome maps of all 12,750 genes aligned by their ATG sites and k-means clustered into 5 groups. Gene order in each cluster was sorted by increasing length.

C. Box-and-whisker plots show similar distribution of WT growth gene expression for each cluster group.

Figure S3. Comparison of MNase-digested chromatin to sonicated naked DNA.

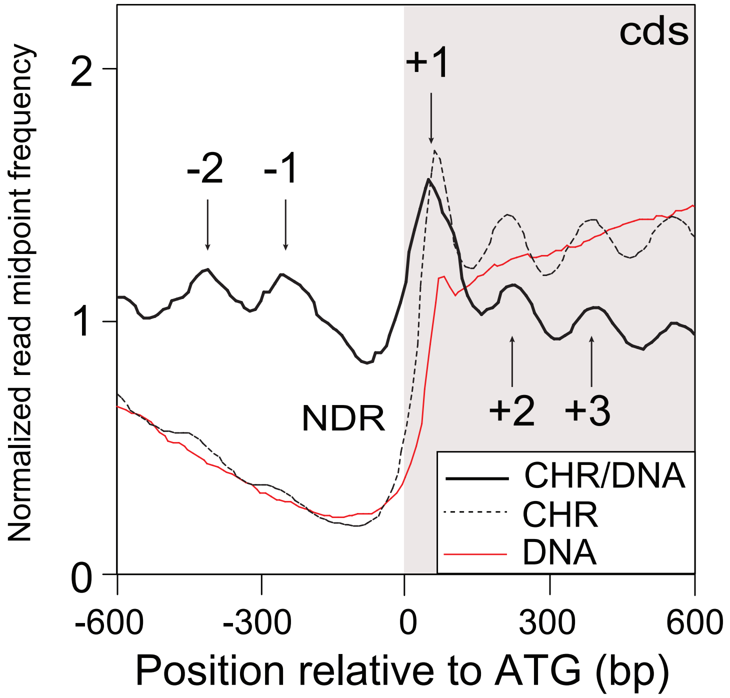

Figure S3. Comparison of MNase-digested chromatin to sonicated naked DNA

Normalized read midpoint frequency distributions for WT chromatin [CHR; dotted line (see Fig. 1A)] were adjusted for sequence mappability by dividing with equivalent control data from sonicated naked (protein-free) WT DNA (DNA; red line) and re-plotted as the ratio (CHR/DNA; thick black line) within 1.2 Kb of flanking chromatin relative to ATG sites of all 12,750 genes. The NDR and positioned nucleosomes upstream and downstream to the NDR are indicated by arrows (see Fig. 1C). The protein coding DNA sequence (cds) region is shaded.

Figure S4. Nucleosomes are remodeled in a subset of genes during WT development.

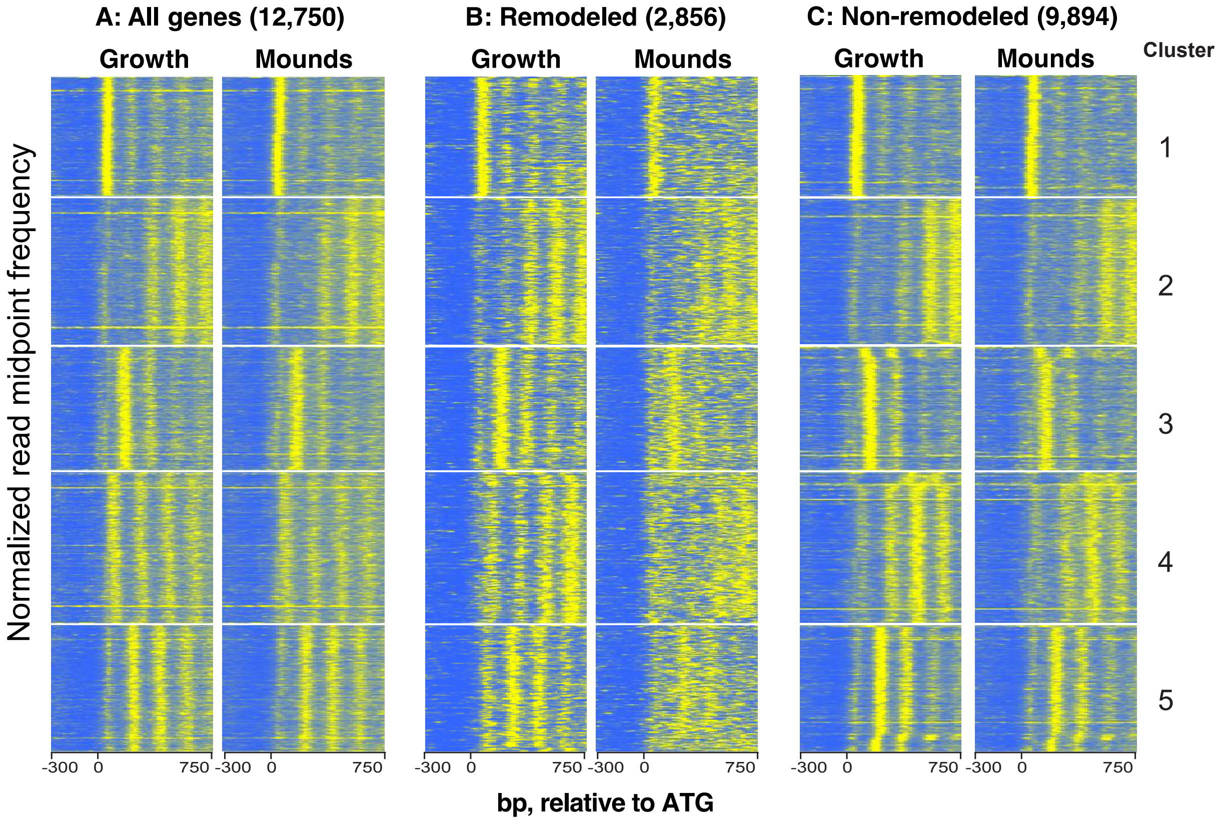

Figure S4. Nucleosomes are remodeled in a subset of genes during WT development.

The nucleosome maps for (**A**) all 12,750 genes, (**B**) the 2,856 developmentally remodeled genes, and (**C**) the 9,894 non-remodeled genes, aligned by their ATG sites, and k-means clustered into 5 groups from both growth and loose mound stage cells. Gene order was first determined for chromatin in growing cells.

Figure S5. Global nucleosome repeat length (NRL) differs in WT between growth and development.

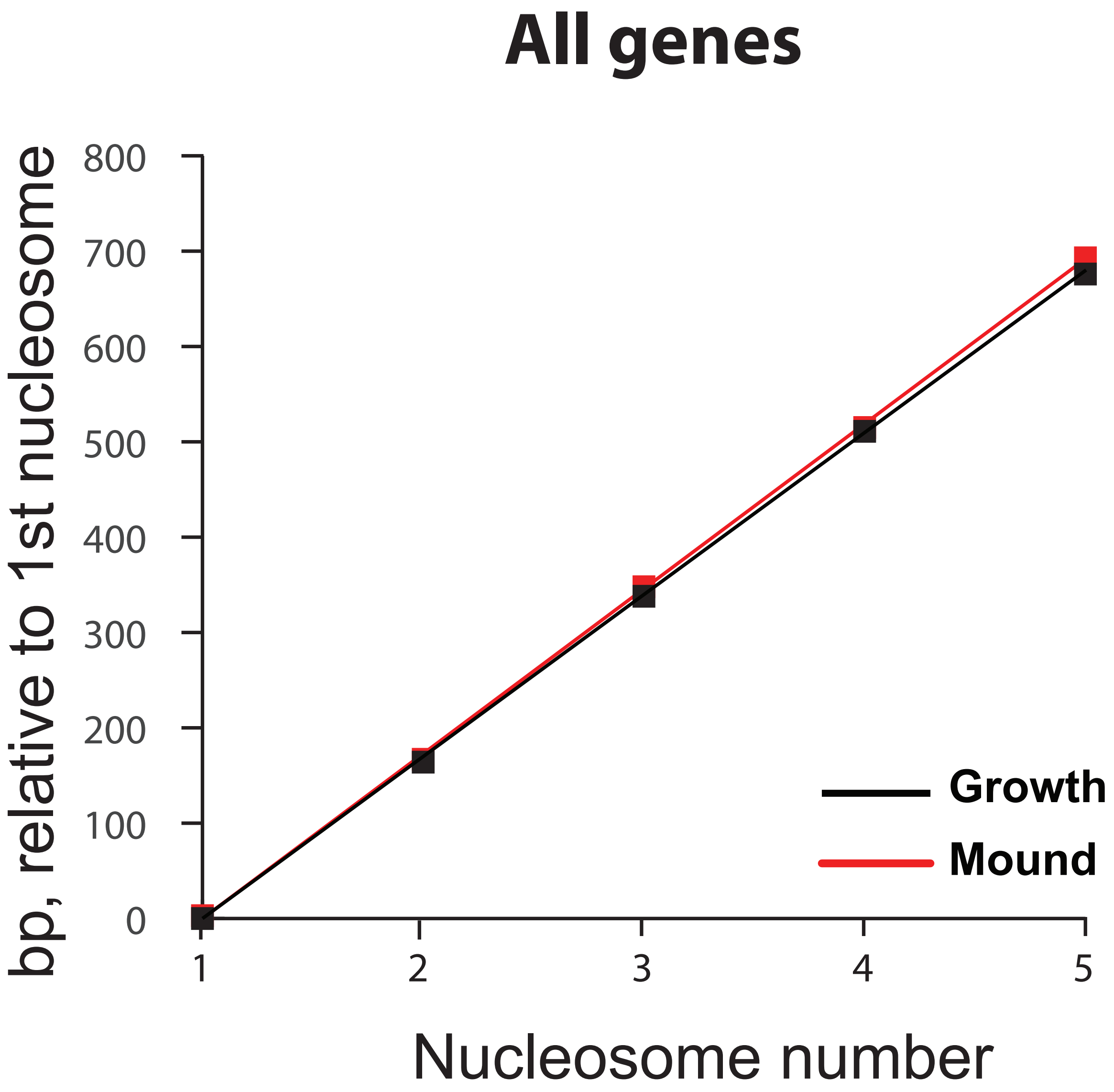

Figure S5. Global nucleosome repeat length (NRL) differs in WT between growth and development.

The average bp position for each nucleosome in growing or loose mound WT cells relative to the position of the first nucleosome in growing WT cells are plotted for all 12,750 genes (see Fig. 2B and Supplementary Table S1). NRLs were determined by linear regression. The NRL in WT growing cells is 170bp (R^2 = 0.9999), whereas the NRL in WT loose mound cells is 173 bp (R^2 = 0.9998).

Figure S6. Developmentally regulated gene expression during WT development.

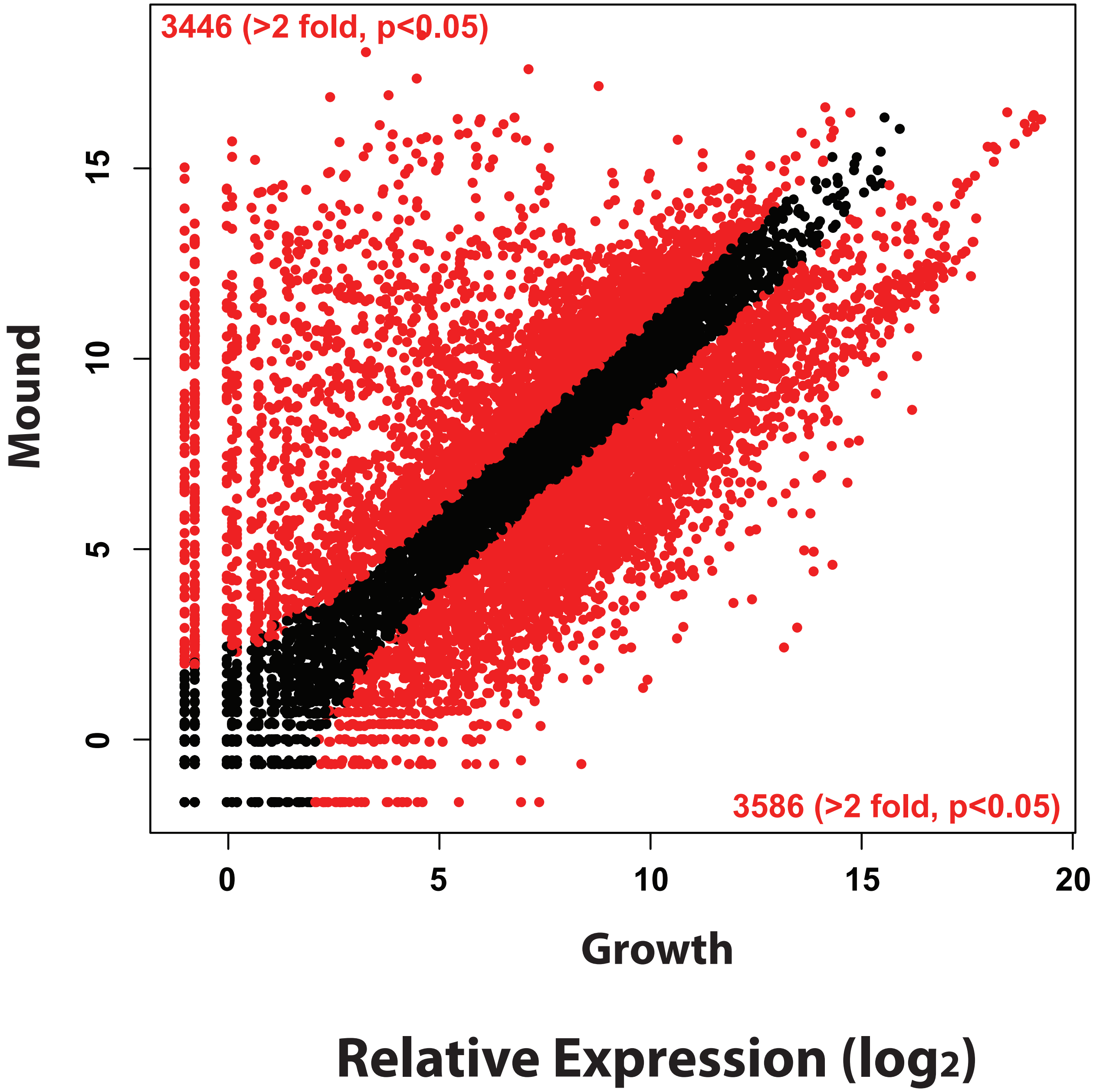

Figure S6. Developmentally regulated gene expression during WT development.

Scatter plots of normalized (FPKM, log2) RNA-seq values for WT cells during growth or loose mound stage. Red values are >2-fold (*p*<0.05) expression differences between the two stages, and black values have <2-fold (*p*<0.05) expression differences.

Figure S7. Nucleosomes are mismodeled in a subset of genes during growth of *chdC*-null cells.

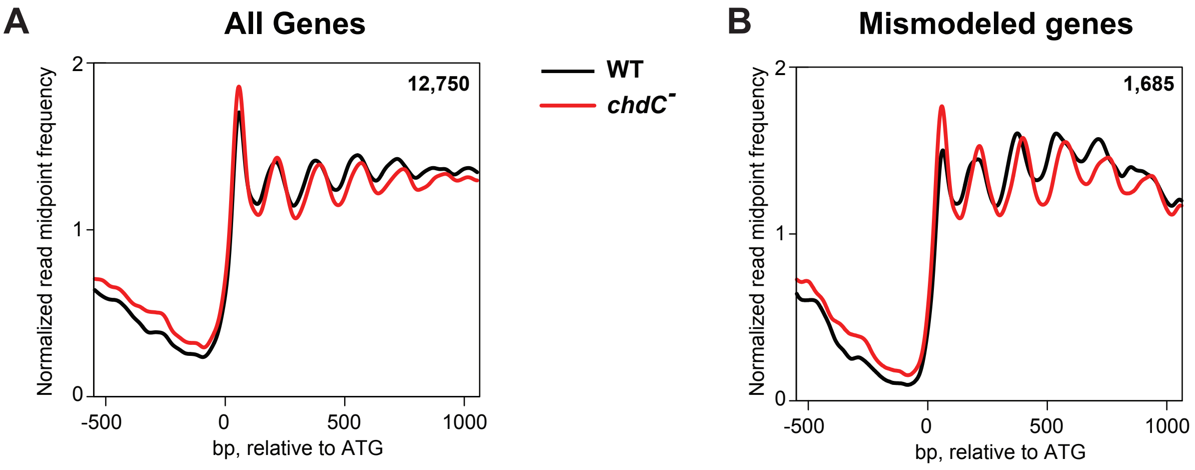

Figure S7. Nucleosomes are mismodeled in a subset of genes during growth of *chdC*-null cells.

A. Mean normalized read midpoint frequencies in growing WT (black) and *chdC*-null (red) cells for all 12,750 genes, aligned to their ATG sites.

B. Mean normalized read midpoint frequencies in growing WT (black) and *chdC*-null (red) cells for the 1,685 mismodeled genes in growing *chdC*-null cells, aligned to their ATG sites.

Figure S8. Nucleosome repeat length (NRL) is lengthened in a subset of genes in *chdC*-null cells during growth and development.

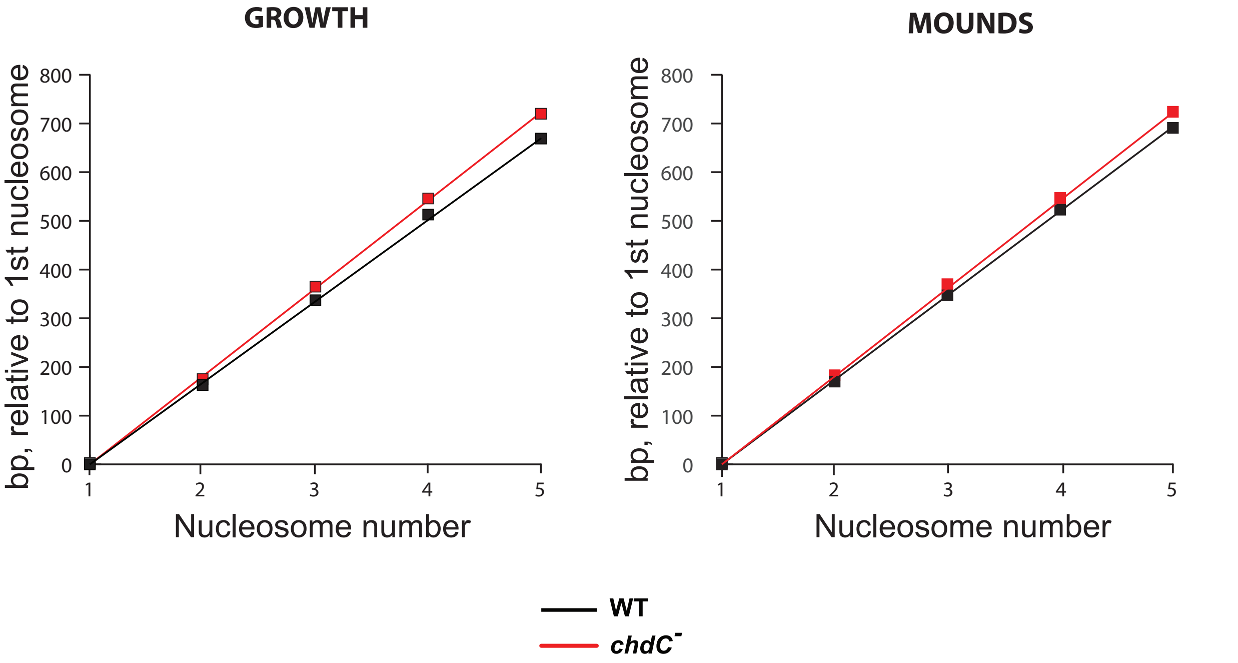

Figure S8. Nucleosome repeat length (NRL) is lengthened in a subset of genes in *chdC*-null cells during growth and development.

The average bp position for each nucleosome in WT (black) or *chdC*-null (red) cells relative to the position of the first nucleosome in WT cells are plotted for genes that are mismodeled in growing or loose mound *chdC*-null cells. Nucleosome repeat length (NRL) was determined by curve fit analyses. In WT cells, the nucleosome repeat length of genes classified as mismodeled matches that of the whole genome, ~170 bp (R^2 = 0.9998) for growth and ~173 bp (R^2 = 0.9999) for the loose mound (see Figs. 4B and 4E and Supplementary Tables S2 and S3). The nucleosome repeat length of mismodeled genes in *chdC*-null cells is ~181 bp (R^2 = 0.9996), regardless if cells are growing or developed (see Figs. 4C and 4E and Supplementary Tables S2 and S3).

Figure S9. The *chdC*-null phenotype correlates with large changes in gene expression during growth and development.

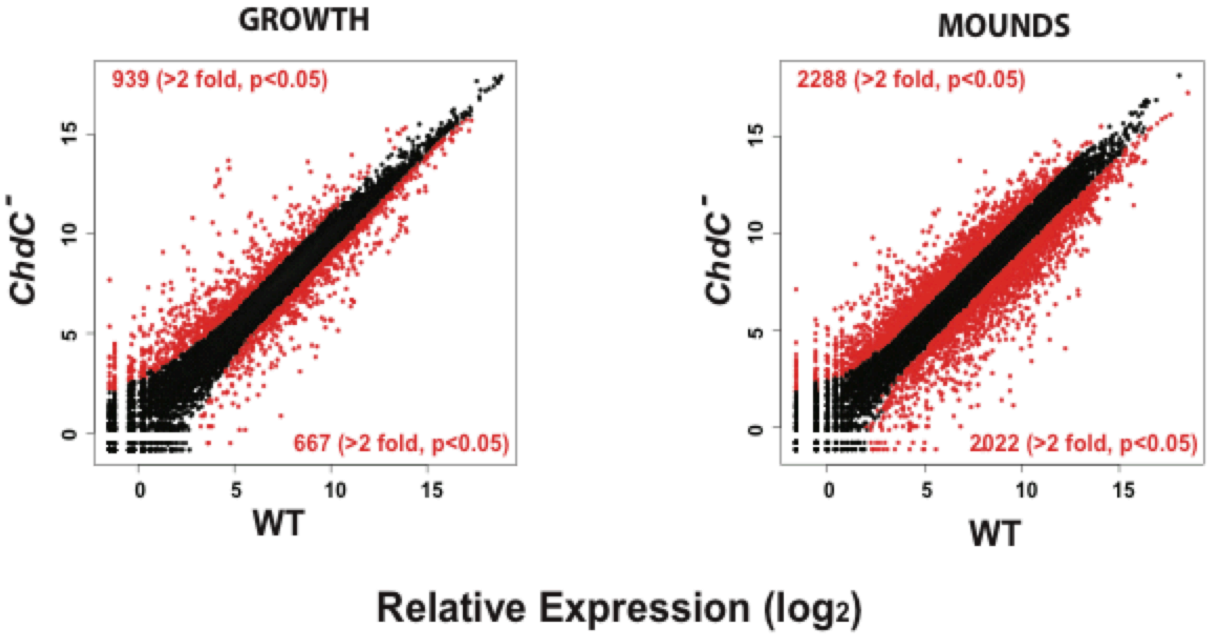

Figure S9. The *chdC*-null phenotype correlates with large changes in gene expression during growth and development.

Scatter plots of normalized (FPKM, log2) RNA-seq values for WT and *chdC*-null cells during growth or loose mound stage. Red values are >2-fold (p<0.05) expression differences between WT and *chdC*-nulls, and black values have <2-fold (p<0.05) expression differences.

Figure S10. Reproducibility of RNA-seq data for WT and *chdC*-null cells.

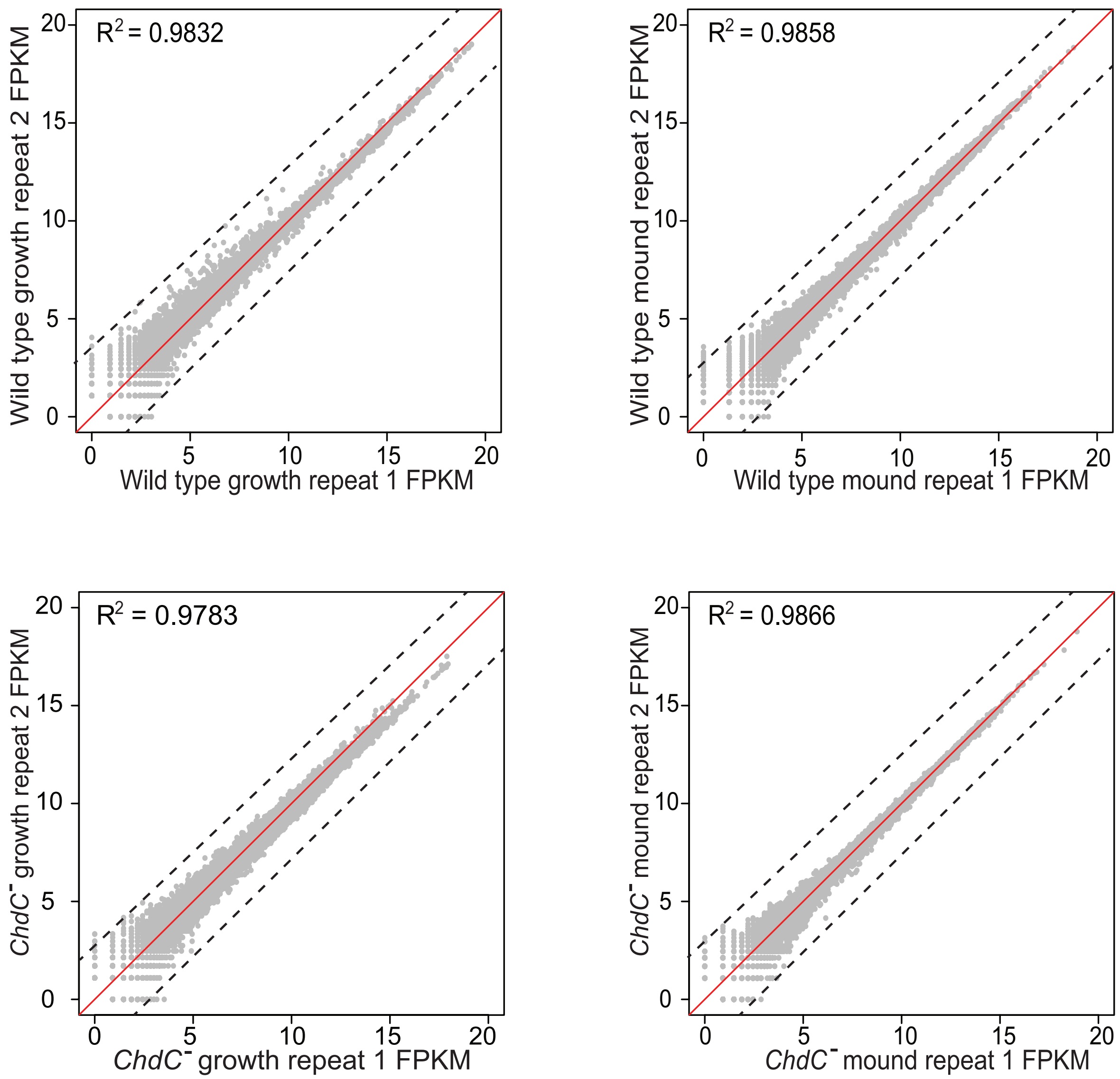

Figure S10. Reproducibility of RNA-seq data for WT and *chdC*-null cells.
Scatter plots of normalized (FPKM, log2) RNA-seq values for biological repeats of WT and *chdC*-null cells during growth or loose mound stage. Dotted lines indicate boundary of > 2-fold difference.

Supplementary Table S1

Nucleosome Spacer Differences: Loose Mound/Growth, WT Cells

STAGE	CHR. CLASS	GENE #	NRL *	R^2 FIT *	ΔLM/Grw ^
Grw (Exp.A)	All	12,750	170 bp	0.9998	0 bp ^
Grw (Exp.B)	All	12,750	170 bp	0.9999	
Grw	All	12,750	170 bp	0.9999	+3 bp
LM	All	12,750	173 bp	0.9998	
Grw	Non-ReMod	9,894	171 bp	0.9999	+3 bp
LM	Non-ReMod	9,894	174 bp	0.9998	
Grw	ReMod	2,856	169 bp	0.9997	+3 bp
LM	ReMod	2,856	172 bp	0.9995	

Grw, growth; LM, loose mound; Chr. Class, chromatin class; ReMod, genes remodeled in WT from growth to loose mound; Exp. A & Exp. B, separate biological/technical experimental replicates (see Supplemental Fig. S1).

* NRL, Nucleosome Repeat Length (bp distance between nucleosome dyad centers), derived from plots aligned to first nucleosome for growth. Value is the mean repeat distance calculated between the first 5 nucleosomes present within the gene coding sequence by regression analysis (see Supplemental Figure S5); R^2 FIT, R^2 value for linear regression analysis.

^ ΔLM/Grw, NRL bp difference between growth and loose mound stage. For Grw Exp. A & Exp. B, NRL bp difference is between separate biological/technical experimental replicates for growing cells.

Supplementary Table S2

Nucleosome Spacer Differences: *chdC*$^{-}$/WT Cells, Growth

STRAIN	CHR. CLASS	GENE #	NRL *	R^2 FIT *	Δ*chdC*$^{-}$ /WT ^
**WT**	**All**	**12,750**	**170 bp**	**0.9999**	**+5 bp**
chdC $^{-}$	**All**	**12,750**	**175 bp**	**0.9998**	
**WT**	**Non-MisMod**	**11,065**	**169 bp**	**0.9998**	**+4 bp**
chdC $^{-}$	**Non-MisMod**	**11,065**	**173 bp**	**0.9999**	
**WT**	**MisMod**	**1,685**	**169 bp**	**0.9998**	**+12 bp**
chdC	**MisMod**	**1,685**	**181 bp**	**0.9996**	

Chr. Class, chromatin class; MisMod, genes mismodeled in *chdC*$^{-}$ cells compared to WT at growth. Non-MisMod, genes that are not mismodeled in *chdC*$^{-}$ cells compared to WT at growth.

* NRL, Nucleosome Repeat Length (bp distance between nucleosome dyad centers), derived from plots aligned to first nucleosome for growth. Value is the mean repeat distance calculated between the first 5 nucleosomes present within the gene coding sequence by regression analysis (see Supplemental Figure S8); R^2 FIT, R^2 value for linear regression analysis.

^ Δ LM/Grw, repeat length difference between WT and in *chdC*$^{-}$ cells during growth. The same number differences are obtained when aligned to the ATG.

Supplementary Table S3

Nucleosome Spacer Differences: *chdC*⁻/WT Cells, Loose Mound

STRAIN	CHR. CLASS	GENE #	NRL *	R^2 FIT *	Δ*chdC*⁻ /WT ^
**WT**	**All**	**12,750**	**174 bp**	**0.9999**	**+3 bp**
***chdC*⁻**	**All**	**12,750**	**177 bp**	**0.9999**	
**WT**	**Non-MisMod**	**10,786**	**172 bp**	**0.9999**	**+3 bp**
***chdC*⁻**	**Non-MisMod**	**10,786**	**175 bp**	**0.9999**	
**WT**	**MisMod**	**1,984**	**173 bp**	**0.9999**	**+8 bp**
chdC	**MisMod**	**1,984**	**181 bp**	**0.9996**	

Chr. Class, chromatin class; MisMod, genes mismodeled in *chdC*⁻ cells compared to WT at the loose mound stage. Non-MisMod, genes that are not mismodeled in *chdC*⁻ cells compared to WT at the loose mound stage.

* NRL, Nucleosome Repeat Length (bp distance between nucleosome dyad centers), derived from plots aligned to first nucleosome for growth. Value is the mean repeat distance calculated between the first 5 nucleosomes present within the gene coding sequence by regression analysis (see Supplemental Figure S8); R^2 FIT, R^2 value for linear regression analysis.

^ ΔLM/Grw, repeat length difference between WT and in *chdC*⁻ cells at the loose mound stage. The same number differences are obtained when aligned to the ATG.
